# Association between low-density lipoprotein cholesterol and frailty in adults aged ≥70 years: a cross-sectional study from Beijing, China

**DOI:** 10.3389/fendo.2026.1789174

**Published:** 2026-03-19

**Authors:** Wenjing Liu, Yuelin Yu, Shanshan Chen, Xin Ren, Yuting Kang, Xiaojuan Zhou

**Affiliations:** 1 Department of Healthcare, Beijing Hospital, National Center of Gerontology; Institute of Geriatric Medicine, Chinese Academy of Medical Sciences, Beijing, China; 2 Office of National Clinical Research for Geriatrics, Department of Scientific Research, Beijing Hospital, National Center of Gerontology; Institute of Geriatric Medicine, Chinese Academy of Medical Sciences, Beijing, China

**Keywords:** cholesterol, cross-sectional study, frailty, low-density lipoprotein cholesterol, older adults

## Abstract

**Background:**

Frailty becomes increasingly prevalent with advancing age and is influenced by multifactorial physiological and pathological processes. This study aimed to assess the current prevalence of frailty among adults aged ≥70 years and to investigate the association between low-density lipoprotein cholesterol (LDL-C) levels and frailty.

**Methods:**

A cross-sectional study was conducted on 218 adults aged 70 and above in Beijing, China, collecting data on their sociodemographic characteristics, lifestyle, comorbidities, and peripheral blood biomarkers. The FRAIL scale was used to measure frailty, and the association between LDL-C and frailty was examined using exploratory analyses that employed ordinal logistic regression, multiple linear regression, and restricted cubic splines (RCS). Stratified analyses were also conducted, dividing participants into subgroups based on polypharmacy, hypertension, diabetes mellitus, coronary heart disease, and alcohol status, to examine the association between LDL-C and frailty.

**Results:**

The mean age of participants was 77.5 ± 6.4 years. The prevalence of robust, pre-frailty, and frailty was 20.6%, 69.7%, and 9.6%, respectively. Adjusted ordinal logistic regression revealed a negative association between LDL-C and frailty (OR = 0.667, 95% CI = 0.489 to 0.909, P = 0.010). Multiple linear regression confirmed this association (β = -0.129, 95% CI = -0.245 to -0.012, P = 0.031). According to the RCS curve, the non-linear relationship between LDL-C and the level of frailty was not significant (P = 0.639). Stratified analyses demonstrated that LDL-C was significantly negatively associated with frailty in non-drinkers and in individuals without hypertension or diabetes mellitus.

**Conclusions:**

Pre-frailty is prevalent among older adults aged ≥70 years, and the relationship between LDL-C and frailty was negative. These findings suggest that individualized lipid management in older adults may need to account for frailty status.

## Introduction

An aging population represents a significant demographic transition, offering both opportunities and challenges. The world's population of adults aged 65 and above is expected to hit 2.2 billion, exceeding the total number of individuals under 18 by the late 2070s. (1). Evidence indicates that human hematopoiesis remains clonally stable and diverse until age 65, after which it deteriorates markedly, with a pronounced acceleration after age 70 (2). This biological phenomenon explains why individuals aged 70 and older are more likely to experience declines in bodily functions. Among adults aged 70 and older, the prevalence of frailty and mortality from non-communicable diseases is significantly elevated compared to those under 70 (3, 4). This leads to reduced physical activity, higher disability rates, and lower quality of life and survival. Understanding these biological and functional declines is critical, as they directly influence frailty prevalence and overall health outcomes in this growing demographic.

With population aging, frailty has emerged as a global public health concern with significant implications for clinical practice and healthcare policy (5). Frailty is a multidimensional geriatric syndrome characterized by increased vulnerability to stressors and declines in physiological reserve (6). This condition increases the risk of adverse outcomes, including falls, hospitalization, and mortality, as well as corresponding increases in healthcare costs (7, 8). Frailty prevalence varies across countries due to differences in demographics, socioeconomic factors, assessment tools, and study settings. In China, the prevalence ranges from 5.9% to 17.4% (9), highlighting the need for population-specific investigations and the substantial challenge that frailty poses to the healthcare system (10). Thus, focused research is urgently needed to clarify the status and determinants of frailty, particularly among the rapidly growing and high-risk population aged 70 and older.

Frailty is associated with a multitude of complex risk factors. Frailty and cardiovascular disease are closely interconnected and can be considered conditions arising from similar underlying mechanisms that expedite their clinical progression (11). Atherosclerosis, the pathological basis of cardiovascular disease, is primarily driven by elevated low-density lipoprotein cholesterol (LDL-C), a well-established causative factor for atherosclerotic cardiovascular disease (12). However, the relationship between LDL-C and frailty remains unclear. A systematic review has reported either no association or a negative correlation between LDL-C levels and frailty incidence (13). Another study has shown a U-shaped relationship between frailty and non-high-density lipoprotein-cholesterol in elderly adults (14). However, a Mendelian randomization study has indicated that genetically lowered lifelong LDL-C is associated with a reduced risk of frailty (15), highlighting the complexity of this relationship. Critically, there is a paucity of evidence specifically for adults aged 70 years and older. This gap limits our understanding of whether the association between LDL-C and frailty in old age is maintained, reversed, or weakened.

Taken together, existing evidence regarding the relationship between LDL-C and frailty remains inconsistent, particularly among adults aged 70 years and older, a population with substantial heterogeneity in health status and comorbidity burden. Clarifying this association in this population may help improve early identification of individuals at risk of frailty and inform more individualized clinical assessment strategies.

## Materials and methods

### Study design and setting

This cross-sectional study was conducted at a Level A tertiary hospital outpatient clinic and a hospital-based physical examination center in Beijing, China, from October 2022 to May 2023. The study was registered with the China Clinical Trial Registry (ChiCTR2200064923).

### Participants and eligibility criteria

Participants were recruited through convenience sampling and enrolled continuously throughout the study period. Inclusion criteria included being 70 years or older and attending a routine health checkup at the hospital outpatient clinic or physical examination center at the study sites. Exclusion criteria were: (1) severe psychiatric illnesses; (2) terminal illnesses with a life expectancy of less than one year; (3) inability to understand or cooperate in completing the structured assessment due to cognitive impairments, language communication disorders, or other reasons.

### Data collection

Following informed consent, all participants underwent a structured assessment. The researchers collected data on sociodemographic, lifestyle, and comorbidity data through face-to-face interviews and a review of electronic medical records. Activities of daily living (ADLs) and frailty were assessed with standardized tools.

Fasting venous blood samples were collected in the morning after an 8-10-hour fast by a registered nurse as part of the participants’ routine health checkup. Key biomarkers were measured using standard automated analyzers as follows: (1) Sysmex XN-9000 Automated Hematology Analyzer: red blood cell count (RBC), white blood cell count (WBC), hemoglobin (HGB), and platelet count (PLT). (2) Cobas^®^ 8000 analyzer (c702 module): high-density lipoprotein cholesterol (HDL-C), low-density lipoprotein cholesterol (LDL-C), and high-sensitivity C-reactive protein (hs-CRP). (3) Abbott Alinity i Chemiluminescence Immunoassay Analyzer: 25-hydroxyvitamin D3 (25-OH-VD3), folic acid (FOL), and vitamin B12 (VitB12).

### Frailty assessment

Frailty, the dependent variable, was evaluated using the FRAIL scale, a 5-item self-reported tool measuring fatigue, resistance, ambulation, illnesses, and weight loss (16). Each item is scored 0 (no) or 1 (yes), resulting in a total score ranging from 0 to 5. Participants were classified as robust (score 0), pre-frailty (scores 1–2), or frailty (scores 3–5). The FRAIL scale was chosen for its brevity, self-report format, and proven validity in identifying frailty among older adults (16–18).

### Covariates

Potential confounders were adjusted for in the analysis, including sociodemographic, lifestyle, clinical status, and functional variables. ADLs were assessed using the Barthel Index, with scores ranging from 0 (complete dependence) to 100 (complete independence). For this analysis, participants were dichotomized into "complete independent" (score = 100) and "mildly dependent" (score = 61–99) groups (19). Definitions and assessment methods for the covariates are provided in **Supplementary Table 1**.

### Statistical analysis

The missing rate for variables in this study ranged from 2% to 10%. After reviewing the data collection process, the missing data were determined to be missing at random. Series mean imputation was applied to variables with less than 5% missing data, providing a stable estimate with minimal impact on the distribution. For variables with 5-10% missing data, regression imputation predicted values based on correlations with other complete variables, thereby preserving the dataset’s internal relationships. Given the cross-sectional design, modest sample size, and low missingness rate (2-10%), single imputation was considered sufficient for this study. Although this method may underestimate variance, sensitivity analyses using complete-case analysis were conducted to assess the stability of the results.

The Kolmogorov-Smirnov test was used to assess the normality of continuous variables. Descriptive statistics were reported as means ± standard deviation (SD) for normally distributed continuous variables and as medians [interquartile range (IQR)] for non- normally distributed variables. Categorical variables were presented as frequencies and percentages. Differences in characteristics across the three frailty groups (robust, pre-frailty, and frailty) were examined using one-way ANOVA or Kruskal-Wallis tests for continuous variables and the chi-squared test for categorical variables. Associations between LDL-C and frailty were analyzed using two primary models: (1) ordered logistic regression with the level of frailty (robust, pre-frailty, frailty) as the ordinal dependent variable, the proportional odds assumption was formally tested using the likelihood ratio test for parallel lines to confirm the model appropriateness; and (2) multiple linear regression with the FRAIL scale score as the dependent variable. Potential confounders identified through univariate analyses and considered clinically relevant were adjusted for in both models. This study also employed RCS to examine the relationship between LDL-C levels and the risk of frailty. An ordinal logistic regression model was constructed with LDL-C as a restricted cubic spline term. Three knots were placed at the 10th, 50th, and 90th percentiles of the LDL-C distribution. The same covariates used in the primary analysis were included for adjustment. Likelihood ratio tests were used to assess the statistical significance of the nonlinear component. Furthermore, stratified analyses were conducted to evaluate whether the association between LDL-C and frailty was modified by the following prespecified factors: polypharmacy, hypertension, diabetes mellitus (DM), coronary heart disease (CHD), and alcohol status.

To assess the reliability of the main results, we conducted two sensitivity analyses. First, the imputation method used in this study might underestimate variance. Therefore, we conducted a complete-case analysis, restricting the sample to participants with no missing data on any variables included in the multivariate model. Second, to minimize the influence of statins, we restricted the sample to participants without CHD and reanalyzed the remaining sample using two regression models (ordinal logistic regression and multiple linear regression). The covariates were identical to those in the main analysis.

All analyses were performed using SPSS (version 20.0) and R (version 4.4.3). A two-tailed P < 0.05 was considered statistically significant.

## Results

### Participant characteristics

The study included 218 adults aged 70 years and older. Based on the FRAIL scale, 45 (20.6%) were robust, 152 (69.7%) were pre-frailty, and 21 (9.6%) were frailty. The mean age was 77.5 ± 6.4 years (range: 70–95), with a clear increasing gradient by frailty severity: robust, 75.0 ± 5.3 years; pre-frailty, 77.6 ± 6.2 years; frailty, 82.4 ± 7.5 years (P < 0.001).

As detailed in **Table 1**, participants with frailty also had a significantly lower body mass index (BMI). Significant inter-group differences were observed for gender, polypharmacy, CHD, physical exercise, self-rated health, and ADLs (P < 0.05). No significant differences were found for marital status, educational level, smoking status, alcohol status, fall history, hypertension, and DM.

**Table 1:** Comparison of the characteristics of participants aged 70 years and older at different levels of frailty (n=218).

Characteristics	Overall (n=218)	Robust (n=45)	Pre-frailty (n=152)	Frailty (n=21)	*F*/χ*²*	P
Age (year)	77.5 ± 6.4	75.0 ± 5.3	77.6 ± 6.2	82.4 ± 7.5	10.527	<0.001
Male	169(77.5)	26(57.8)	129(84.9)	14(66.7)	16.196	<0.001
BMI (kg/m²)	24.66 ± 3.19	24.71 ± 2.78	24.87 ± 3.15	22.95 ± 3.85	3.437	0.034
Marital status					0.180	0.914
Married	203(93.1)	42(93.3)	141(92.8)	20(95.2)		
Divorced/Unmarried/Widowed	15(6.9)	3(6.7)	11(7.2)	1(4.8)		
Educational level					0.839	0.657
High school or below	21(9.6)	5(11.1)	13(8.6)	3(14.3)		
Bachelor’s degree or above	197(90.4)	40(88.9)	139(91.4)	18(85.7)		
Smoking status					2.096	0.351
No	200(91.7)	41(91.1)	138(90.8)	21(100)		
Yes	18(8.3)	4(8.9)	14(9.2)	0(0)		
Alcohol status					3.752	0.153
No	152(69.7)	35(77.8)	100(65.8)	17(81.0)		
Yes	66(30.3)	10(22.2)	52(34.2)	4(19.0)		
Physical Exercise ≥180 min per week					9.162	0.010
No	48(22.0)	10(22.2)	28(18.4)	11(52.4)		
Yes	170(78.0)	35(77.8)	124(81.6)	10(47.6)		
Falls in the last 6 months					0.228	0.892
No	201(92.2)	41(91.1)	141(92.8)	19(90.5)		
Yes	17(7.8)	4(8.9)	11(7.2)	2(9.5)		
Self-rated Health					16.651	<0.001
Fair or Poor	119(54.6)	15(33.3)	86(56.6)	18(85.7)		
Good and above	99(45.4)	30(66.7)	66(43.4)	3(14.3)		
Polypharmacy					23.667	<0.001
No	94(43.1)	33(73.3)	57(37.5)	4(19.0)		
Yes	124(56.9)	12(26.7)	95(62.5)	17(81.0)		
Hypertension					3.795	0.150
No	87(39.9)	22(48.9)	60(39.5)	5(23.8)		
Yes	131(60.1)	23(51.1)	92(60.5)	16(76.2)		
Diabetes Mellitus					0.085	0.958
No	141(64.7)	29(64.4)	99(65.1)	13(61.9)		
Yes	77(35.3)	16(35.6)	53(34.9)	8(38.1)		
Coronary heart disease					11.839	0.003
No	148(67.9)	38(84.4)	101(66.4)	9(42.9)		
Yes	70(32.1)	7(15.6)	51(33.6)	12(57.1)		
Activities of daily living					21.921	<0.001
Mildly dependent	35(16.1)	1(2.2)	24(15.8)	10(47.6)		
Complete independent	183(83.9)	44(97.8)	128(84.2)	11(52.4)		

The comparison of age and body mass index used ANOVA; other data were analyzed with the chi-square test.

Polypharmacy, the concurrent use of five or more medications, including prescription drugs, over-the-counter drugs, and/or traditional and complementary medicines. Activities of daily living is Mildly dependent, the Barthel index’s scores range from 61–99 points. Activities of daily living is Complete independent: the Barthel index’s scores 100 points. BMI, body mass index.

Bold values indicate P < 0.05.

Comparisons of laboratory biomarkers are presented in **Table 2**. Significant differences in RBC, HGB, PLT, LDL-C, and hs-CRP levels across frailty categories were observed (P < 0.05). Notably, LDL-C levels showed an inverse gradient, with the highest levels in the robust group and the lowest in the frailty group. Levels of 25-OH-VD3 and FOL showed a non-significant decreasing trend with increasing frailty. No significant differences were observed for WBC, HDL-C, or VitB12.

**Table 2:** Comparison of laboratory biomarkers in participants aged 70 years and older at different levels of frailty.

Characteristics	Overall(n=218) mean ± SD/m(IQR)	Robust(n=45) mean ± SD/m(IQR)	Pre-frailty(n=152) mean ± SD/m(IQR)	Frailty(n=21) mean ± SD/m(IQR)	*F/H*	P
RBC(×10^12^/L)	4.52 ± 0.47	4.64 ± 0.41	4.53 ± 0.47	4.22 ± 0.46	6.099	**0.003**
WBC(×10^9^/L)	5.85 ± 1.46	6.25 ± 1.47	5.69 ± 1.40	6.10 ± 1.70	2.877	0.058
HGB(g/L)	139.92 ± 14.06	142.11 ± 12.35	140.64 ± 14.00	130.11 ± 14.60	6.152	**0.003**
PLT(×10^9^/L)	200.14 ± 52.83	216.31 ± 54.00	194.30 ± 49.39	207.81 ± 67.40	3.328	**0.038**
HDL-C(mmol/L)	1.42 ± 0.39	1.46 ± 0.35	1.39 ± 0.39	1.53 ± 0.48	1.599	0.205
LDL-C(mmol/L)	2.61 ± 0.99	3.10 ± 1.08	2.50 ± 0.96	2.38 ± 0.68	7.366	**0.001**
hs-CRP*(mg/L)	0.58(0.71)	0.76(0.90)	0.50(0.59)	0.59(1.01)	7.355	**0.025**
25-OH-VD3*(ng/mL)	20.25(12.45)	18.3(12.75)	20.25(13.70)	23.7(17.70)	3.318	0.190
FOL*(ng/mL)	11.20(8.61)	9.60(8.81)	11.25(8.93)	12.3(9.54)	0.314	0.855
VitB12(pg/mL)	627.58 ± 289.18	618.96 ± 276.58	636.63 ± 300.13	580.61 ± 236.23	0.369	0.692

* indicates that the data were not consistent with normal distribution, using the Kruskal-Wallis Test. The rest were consistent with normal distribution, using ANOVA.

RBC, red blood cell; WBC, white blood cell; HGB, hemoglobin; PLT, Platelets; HDL-C, high-density lipoprotein cholesterol; LDL-C, low-density lipoprotein cholesterol; hs-CRP, high-sensitivity C-reactive protein; 25-OH-VD3, 25-hydroxyvitamin D3; FOL, folate; VitB12, Vitamin B12.

Bold values indicate P < 0.05.

### Multivariable analysis of LDL-C and frailty

Analysis of the association between LDL-C and frailty using two regression models (**Table 3**). The proportional odds assumption was formally tested with the likelihood ratio test for parallel lines and was not violated (χ*²* = 13.58, df = 8, P = 0.093), confirming the appropriateness of the ordinal logistic regression model. The ordinal logistic regression results showed that, after adjusting for variables such as age, physical exercise, self-rated health, ADLs, BMI, HGB, and hs-CRP, LDL-C levels were significantly negatively associated with the level of frailty (OR = 0.667, 95% CI = 0.489 to 0.909, P = 0.010). Multiple linear regression analysis also indicated a significant negative association between LDL-C and the frailty score (β = -0.129, 95% CI = -0.245 to -0.012, P = 0.031). The results from both models consistently support a negative correlation between LDL-C and frailty. To further explore the potential complex relationship, a dose-response analysis was conducted using RCS. After adjusting for age, BMI, HGB, hs-CRP, physical exercise, ADLs, and self-rated health, the nonlinear test was not significant (P = 0.639). The specific trend is shown in **Supplementary Figure 1**.

**Table 3:** Multivariable analysis of frailty in participants aged 70 years and older.

Variables		Ordinal logistic regression		Multiple linear regression	
		OR (95%CI)	P	β (95%CI)	P
Age		1.061(1.003,1.123)	**0.040**	0.022(0.002, 0.042)	**0.034**
Physical Exercise ≥180min per week	No	1.567(0.738,3.329)	0.242	-0.306(-0.581, -0.031)	**0.030**
	Yes	1.00		0.00	
Self-rated Health	Fair or Poor	3.070(1.608,5.860)	**0.001**	-0.418(-0.646, -0.190)	**<0.001**
	Good and above	1.00		0.00	
ADLs	Mildly dependent	3.737(1.422,9.822)	**0.007**	-0.482(-0.821, -0.142)	**0.006**
	Complete independent	1.00		0.00	
BMI		0.947(0.858,1.046)	0.283	-0.011(-0.047, 0.025)	0.543
HGB		0.996(0.972,1.020)	0.732	-0.003(-0.012, 0.006)	0.553
LDL-C		0.667(0.489,0.909)	**0.010**	-0.129(-0.245, -0.012)	**0.031**
hs-CRP		0.937(0.836,1.050)	0.263	-0.012(-0.056, 0.033)	0.608

ADLs, activities of daily living; BMI, body mass index; HGB, hemoglobin; LDL-C, low-density lipoprotein cholesterol; hs-CRP, high-sensitivity C-reactive protein.

Bold values indicate P < 0.05.

**Figure 1:**
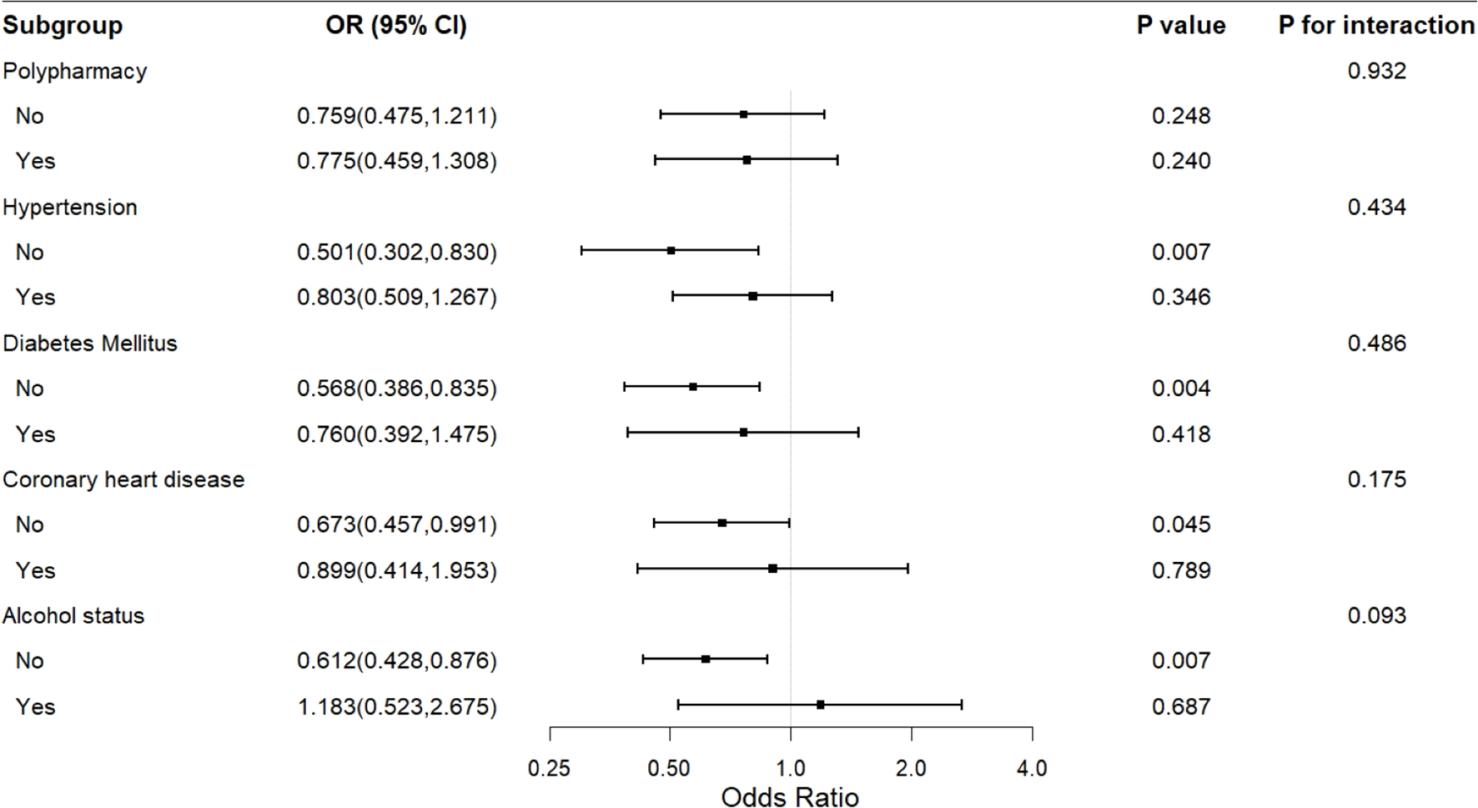
Forest plot of stratified analysis of the association between LDL-C and frailty by ordinal logistic regression.

**Figure 2:**
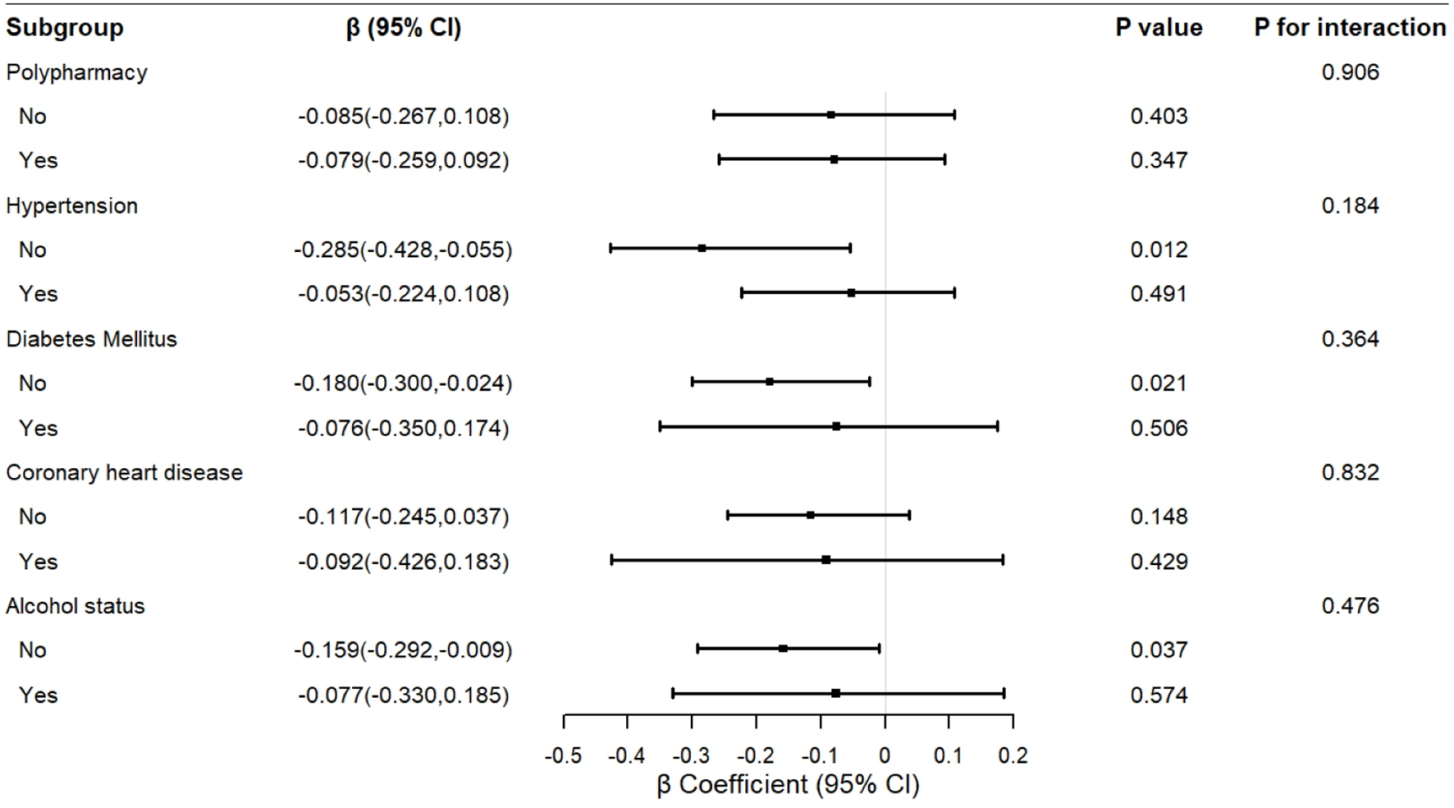
Forest plot of stratified analysis of the association between LDL-C and frailty by multiple linear regression.

Stratification analysis of the relationship between LDL-C and frailty. The exploratory stratified analysis showed that the negative correlation between LDL-C and frailty was more pronounced in the clinical subgroups. The ordinal logistic regression analysis indicated that among participants without hypertension (OR = 0.501, 95% CI = 0.302 to 0.830, P = 0.007), without DM (OR = 0.568, 95% CI = 0.386 to 0.835, P = 0.004), and without alcohol intake (OR = 0.612, 95% CI = 0.428 to 0.876, P = 0.007), LDL-C was significantly negatively correlated with the level of frailty. The multiple linear regression analysis further supported this trend, and in these subgroups, LDL-C was also significantly negatively correlated with the frailty score (P < 0.05). However, in subgroups with the corresponding diseases or alcohol status, the association did not reach statistical significance. The interaction analysis showed no statistically significant difference in the association across subgroups (all P for interaction > 0.05), and given the small sample size of the frail group (n = 21), this subgroup finding should be interpreted as exploratory and hypothesis-generating rather than evidence of effect modification. The detailed results are shown in **Figures 1**, **2**.

### Sensitivity analysis

To assess the robustness of the primary findings, two sensitivity analyses were conducted. First, to evaluate the potential impact of missing-data imputation, a complete-case analysis was performed, excluding participants with any missing data on variables in the multivariable model (n = 15). Results aligned with the primary analysis: LDL-C remained significantly negatively associated with frailty in both the ordinal logistic regression (OR = 0.637, 95% CI = 0.461 to 0.881, P = 0.006) and the multiple linear regression (β = -0.149, 95% CI = -0.253 to -0.022, P = 0.020). This suggests that the imputation methods did not substantially bias the outcomes. Second, after excluding participants with CHD (n = 70), the model was refitted. Results showed that in the ordinal logistic regression, LDL-C remained significantly negatively associated with frailty (OR = 0.673, 95% CI = 0.457 to 0.991, P = 0.045). In the multiple linear regression, this association did not reach statistical significance (β = -0.104, 95% CI = -0.245 to 0.037, P = 0.148). These findings suggest that the negative association between LDL-C and frailty is robust to exclusion of CHD patients when frailty is analyzed as an ordinal outcome, but less stable when analyzed as a continuous score, possibly reflecting reduced statistical power in this restricted sample (n = 148). Detailed results are presented in **Supplementary Table 2**.

## Discussion

### High prevalence of pre-frailty

This cross-sectional study found that 9.6% of adults aged ≥70 years were in the frailty group, and 69.7% were in the pre-frailty group. The pre-frailty rate observed here was higher than in several previous studies, likely attributable to the older mean age of participants (77.5 vs. 72.3/71.5 years) and to the recruitment of all participants from Beijing, a region characterized by rapid population aging (20, 21). Nevertheless, the overall frailty prevalence of 9.6% aligns with established epidemiological trends, which typically range from 4% to 26% in older age groups (22). Pre-frailty, defined as a state of diminished physiological reserve and heightened stress vulnerability that precedes overt frailty (23), represents a substantial yet under-recognized public health burden. Its high prevalence signals both a significant public health challenge and a critical window for preventive intervention. Currently, this window is often overlooked, as clinical practice prioritizes symptomatic disease management. Given that an estimated 4-7% of robust or pre-frailty older adults transition to frailty each year (24), a dual-strategy approach is warranted: first, to mitigate disability in those already frail; and second, and more critically, to prevent progression from pre-frailty to frailty through early identification and tailored interventions.

### Mechanistic interpretation of the ‘cholesterol paradox’

Current lipid management guidelines generally advocate a “lower is better” strategy for LDL-C (25–27). However, this study found a negative correlation between LDL-C levels and frailty among adults aged 70 and older, and this association persisted even after excluding individuals with CHD, consistent with the “cholesterol paradox”. Previous studies have shown that the relationship between LDL-C and mortality is heterogeneous: a systematic review found that in the elderly population, LDL-C was either unrelated to or negatively correlated with mortality (13), whereas in healthy elderly individuals, the two were in a U-shaped relationship (28). An extensive Chinese cohort study found that the optimal LDL-C range of 100–159 mg/dL was most appropriate for all-cause mortality risk (29). Patients with high baseline LDL-C hypercholesterolemia have a lower risk of adverse events (30), and in patients with liver cirrhosis, although elevated LDL-C is associated with cardiovascular events, too low LDL-C increases the risk of all-cause mortality (31). Although these studies did not examine frailty status, their common conclusion is that LDL-C in the elderly may shift from a traditional cardiovascular risk factor to a marker of overall health status. It should be emphasized that this study is cross-sectional and cannot establish a causal relationship. The following mechanistic discussions aim to provide possible biological explanations for the observed association, rather than to make causal inferences.

Inflammation-driven lipid remodeling: Chronic inflammation may be the common pathway linking LDL-C and frailty. Although hs-CRP was included as a covariate in this study, it did not reach statistical significance, likely reflecting the limitation of a single biomarker in capturing the complex, chronic low-grade inflammatory state characteristic of frailty. This does not preclude an important role for inflammation, given evidence that inflammatory biomarkers often reflect subclinical vulnerability more accurately than lipid parameters in metabolically heterogeneous populations (32). Specifically, pro-inflammatory cytokines can drive muscle catabolism and functional decline in frailty (33) and upregulate the LDL receptor on hepatocytes, increasing hepatic LDL uptake and the secretion of cholesterol into bile, thereby lowering blood LDL-C (34). The inflammatory process itself increases cholesterol catabolism, promotes LDL receptor expression, and promotes oxidation. Oxidized LDL, in turn, promotes inflammation and has adverse health effects (35). This provides a possible explanatory framework for the ‘cholesterol paradox’: inflammation simultaneously remodels lipid metabolism and degrades musculoskeletal function, rendering low LDL-C a concomitant phenomenon rather than an independent protective factor. Functional depletion of cholesterol: In older adults, LDL-C may shift from a “risk factor” to a "survival resource". Cholesterol (including LDL-C) contributes to host defense by neutralizing bacterial toxins and forming a barrier against infection (36). Moreover, LDL-C contributes to endothelial damage and repair through oxidative stress in endothelial cells (37) and stimulates the proliferation of CD34-positive cells to maintain vascular homeostasis (38). Cholesterol is a precursor of steroid hormones, which are closely linked to metabolism and immune response in older adults. Lower cholesterol levels may exacerbate age-related hormone deficiencies (39). Therefore, low LDL-C levels may indicate an increased risk of infection, cumulative vascular damage, and metabolic-immune dysregulation, reflecting insufficient multidimensional physiological reserves for defense, metabolism, and vascular homeostasis.

Nutritional vulnerability: Malnutrition provides a third explanatory pathway for the cholesterol paradox. Insufficient energy and protein intake can impair LDL-C synthesis, while accompanying frailty-related problems can accelerate cholesterol catabolism. This pattern of nutritional metabolism is strongly illustrated in the research by Wang et al. (40), which included 41229 patients with CHD. Among these patients, 90.3% with LDL-C levels below 1.8 mmol/L were malnourished. Notably, after adjusting for nutritional status (CONUT score), the initially observed association of “low LDL-C with higher mortality” reversed. This significant change suggests that low LDL-C levels may reflect underlying malnutrition, especially in older adults, where protein-energy malnutrition and frailty often coexist.

### Survival bias and population heterogeneity

Although this study is an exploratory cross-sectional analysis. The stratified results provide preliminary insights into the complex relationship between LDL-C and frailty. We acknowledge that these results may be affected by survival bias and population heterogeneity. The elderly population who have lived to age 70 has already undergone natural selection. Individuals who have maintained a low LDL-C level over the long term may represent an aging phenotype with a unique genetic background or lifestyle. Notably, the stratified analysis shows that the relationship between LDL-C and frailty varies across subgroups, particularly in relatively healthy groups, including those without hypertension, diabetes, or alcohol consumption. In the relatively healthy elderly population, LDL-C may more accurately reflect the physiological state related to aging (41). In contrast, in populations with chronic diseases, LDL-C levels become inconsistent due to disease effects and multiple medications. Notably, the frailty group in this study had only 21 participants, which greatly limits statistical power and weakens the reliability of the subgroup analysis results. Therefore, the results from the stratified analysis should be interpreted as exploratory and hypothesis-generating rather than as evidence of a difference in association between clinical subgroups.

### Residual confounding

Unmeasured statin use may confound the observed association. We addressed this in a sensitivity analysis by excluding participants with CHD, a proxy for high statin use; the persistence of the negative correlation suggests that statin use alone is unlikely to fully account for our findings. This is supported by prior evidence: in ACS patients, lower admission LDL-C was associated with a poor prognosis independent of statin therapy (42), with frailty and inflammation identified as key drivers; similarly, in heart failure, lower LDL-C was associated with mortality in non-diabetic patients despite statin use (43). Collectively, these findings suggest that the LDL-C-frailty relationship is not merely a pharmacological artifact. However, an alternative explanation warrants consideration: statins may directly contribute to frailty. A UK Biobank study demonstrated dose-dependent associations between continuous statin use and accelerated declines in grip strength and lean mass (44). Our exclusion strategy only partially addresses this concern, as it does not capture primary prevention statin use in older adults without CHD. Therefore, residual confounding by unmeasured lipid-lowering therapy cannot be excluded.

### Clinical significance

From a clinical perspective, our findings suggest that extremely low LDL-C levels in adults aged 70 and older may warrant cautious interpretation, particularly in those at risk of frailty. When formulating lipid management strategies, it may be reasonable to consider overall health status and to balance potential benefits and risks on an individual basis. Rather than relying solely on lipid targets, a comprehensive geriatric assessment that includes functional status and comorbidity burden may provide a more appropriate framework for shared decision-making in older adults. Further research is needed to determine whether incorporating frailty assessment into lipid management improves clinical outcomes.

### Limitations

This study has several limitations that should be considered when interpreting the findings. First, its cross-sectional design precludes establishing causal relationships between LDL-C and frailty. The observed negative association, while robust in our adjusted models, may be influenced by reverse causality or unmeasured confounding. Second, the modest sample size (n=218), particularly the small number of frail participants (n=21), may limit statistical power and reduce the precision of effect estimates in multivariable analyses for this subgroup. Consequently, findings related to the frailty group should be interpreted with caution. Future large-scale, prospective cohort studies are warranted to confirm the temporal sequence and generalizability of this association. Third, the FRAIL scale primarily focuses on the physical aspects of frailty and does not capture cognitive impairment, nutritional status, or psychosocial factors—important dimensions of frailty. Different frailty assessment tools emphasize different dimensions, which may influence the observed association. It remains unclear whether tools that incorporate objective physical measures or multidimensional deficits would yield similar findings. Future studies should consider employing more comprehensive frailty assessment tools to validate our results. Fourth, our study lacked direct data on the use of lipid-lowering medications, including statins. While excluding CHD patients partially addresses this concern, some participants without CHD may still receive statins for primary prevention. This residual confounding could bias the observed association between LDL-C and frailty. Future studies should record and adjust for lipid-lowering pharmacotherapy. Fifth, for missing-value imputation, we employed series mean and regression imputation. Compared with multiple imputation, this approach may underestimate variance. However, the low missing rate (2-10%) and consistent sensitivity analyses mitigate this concern. Finally, as a single-center study conducted in Beijing, the findings may not be fully generalizable to older adults in other geographic, ethnic, or healthcare settings. Multicenter studies involving diverse populations are needed to validate our results.

## Conclusion

In this study of adults ≥ 70 years, pre-frailty was highly prevalent, and LDL-C was negatively correlated with the level of frailty. Given the cross-sectional design of this study, these findings do not support changes to current clinical guidelines without prospective evidence. In the future, when developing individualized lipid management strategies for older adults, it may be necessary to reconsider frailty as part of a comprehensive assessment, rather than focusing solely on LDL-C levels. Large-scale prospective studies are needed to verify the causal relationship and the generalizability of this association, and to explore lipid management pathways stratified by frailty.

## Data Availability

The datasets presented in this article are not readily available because The original contributions presented in the study are included in the article/**Supplementary Material**. Further inquiries can be directed to the corresponding author. Requests to access the datasets should be directed to bjyybjylbhl@163.com.

## Supplementary material

Supplementary Figure 1 Association of LDL-C with frailty risk modeled using restricted cubic spline analysis.
