# Supplementary material for: Association between low-density lipoprotein cholesterol and frailty in adults aged ≥70 years: a cross-sectional study from Beijing, China
Source: Front Endocrinol (Lausanne). 2026 Mar 19;17:1789174. doi: 10.3389/fendo.2026.1789174 (PMC13043368; doi:10.3389/fendo.2026.1789174)
Supplement: Supplementary file 2 [file Table1.pdf]

Supplementary Table 1. Definitions and Assessment Methods of Covariates

Variable	Type	Categories / Definition	Assessment Method
**Socio-demographic**			
Age	Continuous	Years	Self-report
Gender	Categorical	Male, Female	Self-report
Educational level	Categorical	High school or below, Bachelor degree or above	Self-report
Marital status	Categorical	Married, Other (divorced/unmarried/widowed)	Self-report
**Lifestyle & Health Behavior**			
Smoking status	Categorical	No, Yes	Self-report
Alcohol status	Categorical	No, Yes	Self-report
Physical Exercise ≥180min per week	Categorical	No, Yes	Self-report
**Clinical Status**			
Body mass index (BMI)	Continuous	kg/m^2	Measured
Polypharmacy	Categorical	No, Yes	Medication review
Hypertension	Categorical	No, Yes	Physician diagnosis (EMR)
Diabetes Mellitus	Categorical	No, Yes	Physician diagnosis (EMR)
Coronary heart disease	Categorical	No, Yes	Physician diagnosis (EMR)
Falls in the last 6 months	Categorical	No, Yes	Self-report
Self-rated health	Categorical	Fair or poor, Good and above	Self-report
**Functional Status**			
Activities of Daily Living (ADLs)	Categorical	Mildly dependent, Completely independent	Barthel Index
