# Supplementary material for: Association between low-density lipoprotein cholesterol and frailty in adults aged ≥70 years: a cross-sectional study from Beijing, China
Source: Front Endocrinol (Lausanne). 2026 Mar 19;17:1789174. doi: 10.3389/fendo.2026.1789174 (PMC13043368; doi:10.3389/fendo.2026.1789174)
Supplement: Supplementary file 3 [file Table2.pdf]

Supplementary Table 2. Sensitivity Analysis

Model	LDL-C Coefficient (95% CI)	P
**Primary Analysis (All Participants, n=218)**		
Ordinal Logistic Regression	OR = 0.667 (0.489, 0.909)	0.010
Multiple Linear Regression	β = -0.129 (-0.245, -0.012)	0.031
**Sensitivity Analysis 1(Complete-case analysis, n=203)**		
Ordinal Logistic Regression	OR = 0.637 (0.461, 0.881)	0.006
Multiple Linear Regression	β = -0.149 (-0.253, -0.022)	0.020
**Sensitivity Analysis 2(Excluding CHD, n=148)**		
Ordinal Logistic Regression	OR = 0.673 (0.457, 0.991)	0.045
Multiple Linear Regression	β = -0.104 (-0.245, 0.037)	0.148

Abbreviation: CHD: coronary heart disease; OR: odds ratio; CI: confidence interval.
